# Patient perspectives on atrial fibrillation management and pharmacist roles in outpatient clinics: a qualitative study

**DOI:** 10.1007/s11096-026-02155-7

**Published:** 2026-04-29

**Authors:** Margaret Dennehy, Anna Seoighe, Cathal Cadogan, Kate Burke, Emma Murtagh, Ronan Collins, Derek Hayden, Christine McAuliffe

**Affiliations:** 1 The School of Pharmacy and Pharmaceutical Sciences, Trinity College Dublin, Dublin, Ireland; 2 Pharmacy Department, Tallaght University Hospital, Dublin, Ireland; 3 Atrial Fibrillation and Stroke Services, Tallaght University Hospital, Dublin, Ireland

**Keywords:** Atrial fibrillation, Anticoagulants, Clinical pharmacy, Pharmacist prescribing, Patient perspectives, Shared decision-making

## Abstract

**Introduction:**

Atrial fibrillation (AF) is the most common cardiac arrhythmia and is associated with increased risk of stroke and mortality. Effective management requires both patient engagement and clear understanding of treatment goals yet evidence suggests persistent gaps in knowledge and involvement in care. Pharmacists are increasingly involved in AF clinics, yet little is known about how patients perceive their role, particularly in the context of potential pharmacist prescribing.

**Aim:**

To explore patients’ perspectives on the management of AF and the role of pharmacists in an Irish outpatient AF clinic, including attitudes toward potential pharmacist prescribing.

**Method:**

A qualitative study was conducted using 15 one-to-one semi-structured interviews with adults aged ≥ 65 years attending a specialist AF clinic at Tallaght University Hospital, Dublin, Ireland. Interviews took place between March and May 2025. Participants were purposively sampled to capture a range of clinical and demographic characteristics. Interviews were audio-recorded, transcribed verbatim, and analysed using Braun and Clarke’s six-step reflexive thematic analysis. An inductive approach was adopted. Coding and theme development were conducted iteratively, with a subset of transcripts independently reviewed by supervisors to enhance analytical rigour and consistency.

**Results:**

Five overarching themes were identified relating to understanding of AF, pharmacotherapy, satisfaction with care, perceptions of the pharmacist’s role, and views on pharmacist prescribing. Participants demonstrated variable understanding of AF, with limited awareness of stroke risk despite consistent recognition of anticoagulant therapy. Knowledge of anticoagulant safety, particularly bleeding risk, was strong and well retained, whereas understanding of rate-control medicines was limited. Satisfaction with care was high; however, most participants adopted a passive role in decision-making, expressing trust in healthcare professionals. Pharmacists were frequently misidentified as doctors or nurses, reflecting limited role visibility. Once recognised, pharmacist input, particularly in anticoagulant counselling and medication advice, was highly valued. Views on pharmacist prescribing were mixed: while participants acknowledged pharmacists’ expertise in medicines, many preferred doctors to retain prescribing responsibility due to established trust.

**Conclusion:**

While patients spoke positively about pharmacists’ input, many struggled to identify the pharmacist within the clinic and were unclear about the scope of their role. Gaps in understanding of stroke risk and broader treatment goals highlight the need for structured patient education. As Ireland considers expanding pharmacist prescribing beyond the current common conditions scheme in community pharmacy, strengthening patient education and fostering trust will be essential to optimise pharmacist-led AF services.

**Supplementary Information:**

The online version contains supplementary material available at https://doi.org/10.1007/s11096-026-02155-7.

## Impact Statements

Evaluating patients’ understanding of AF management in an outpatient AF clinic provides insight into the effectiveness of multidisciplinary care and helps identify areas where patient education and support, including pharmacy input, may be strengthened. As pharmacy roles in Ireland expand toward Advanced Specialist Pharmacists and potential pharmacist prescribing, understanding patient trust and expectations is vital to shaping how pharmacists can maximise their impact and help patients get the most from pharmacy-led care.

## Introduction

Atrial fibrillation (AF) is the most common sustained cardiac arrhythmia, associated with significantly increased risks of stroke and mortality [1]. Anticoagulation is a key therapeutic approach to reducing stroke risk in patients with AF and is central to current treatment guidelines [2]. However, AF management extends beyond stroke prevention and also includes rate- and rhythm-control strategies to manage symptoms and improve quality of life as well as lifestyle modification [1]. The complexity of treatment options, lifelong therapy considerations, and associated bleeding risks make patient understanding and engagement essential to effective care [3]. The 2024 European Society of Cardiology (ESC) guidelines emphasise a multidisciplinary approach to AF management, advocating for structured care pathways and active patient involvement [1].

Shared decision-making (SDM) is increasingly recognised as a best practice in AF care [4, 5]. It allows healthcare professionals and patients to make informed decisions together, aligning clinical evidence with the patient’s individual values and preferences. Studies show that SDM improves adherence to anticoagulation and enhances patient satisfaction [6]. However, surveys such as that by the European Heart Rhythm Association (EHRA) revealed that many patients still struggle to understand their diagnosis, with many patients unclear about the purpose of anticoagulation and often unable to identify which of their medications are for AF [4, 7, 8].

Pharmacists are well positioned to address these gaps through medication counselling, safety reviews, and education [9]. Evidence from outpatient and long-term care settings suggests that pharmacist-led interventions improve anticoagulation appropriateness, reduce adverse events, and support more rational prescribing [10]. In Tallaght University Hospital (TUH), Dublin, Ireland, an established weekly AF clinic delivers multidisciplinary care involving stroke consultants, an advanced nurse prescriber (ANP) and a clinical pharmacist [9]. The AF clinic is a specialist service designed primarily for patients aged 65 years and older, accepting referrals for newly diagnosed AF confirmed by electrocardiogram (ECG) [11]. The service provides comprehensive management, including rate/rhythm control and patient education. Clinical pharmacists at TUH are actively involved in reviewing anticoagulant prescriptions, dose optimisation based on renal function, counselling patients on adherence and bleeding risk, and identifying potential drug interactions [9, 11].

Internationally, particularly in Canada, pharmacists have demonstrated value as independent prescribers in AF management [12]. In these models, pharmacist prescribing of anticoagulants in accordance with guideline-based stroke risk assessment has led to improved access to care, increased use of oral anticoagulants, and better stroke prevention outcomes [13, 14]. While pharmacists in Ireland currently do not have independent prescribing rights, this is an area of active policy development. The *Expert Taskforce to Support the Expansion of the Role of Pharmacy* (2024) has recommended a phased introduction of pharmacist prescribing, beginning with 8 defined common conditions being introduced into community pharmacies at the beginning of 2026 and progressing toward full prescriptive authority in both primary and secondary care [15, 16]. Outpatient clinics such as the AF clinic in TUH, where a pharmacist is already embedded within the multidisciplinary team and routinely advises on anticoagulant dose adjustments, could serve as a suitable setting for introducing pharmacist prescribing.

In tandem, new Advanced Specialist Pharmacist (ASP) roles have been introduced across Health Service Executive (HSE) hospitals [17]. These roles formally recognise pharmacists working at an expert level in complex clinical areas, including cardiology. They are expected to lead service initiatives, optimise pharmacotherapy for high-risk patients, and support multidisciplinary teams in line with international advanced practice frameworks [17, 18].

### Aim

This study aimed to explore patients’ perspectives on the management of AF and the current and potential role of pharmacists within this pathway. By examining how patients experience pharmacy services at TUH’s AF clinic and whether they would welcome an expanded role for pharmacists, including prescribing, this work seeks to inform service development and future policy on pharmacist-led care in Ireland.

## Method

### Design

This study used a qualitative, semi-structured interview approach involving patients with AF. Data were analysed using thematic analysis to identify, analyse, and interpret themes within the data collected.The reporting of this study followed the Consolidated Criteria for Reporting Qualitative Research (COREQ) 32-item checklist(Appendix 1) [19].

### Setting

Data for this study were collected from patients attending a weekly specialist AF outpatient clinic at TUH, Dublin, Ireland. This interdisciplinary clinic provides care to patients ≥ 65 years with complex comorbidities, and includes pharmacy, cardiology, and stroke medicine services [9].

### Population

Inclusion criteria comprised adults aged ≥ 65 years with a confirmed diagnosis of AF who had attended the AF clinic at least once and were able to provide informed consent. Participants required sufficient English language proficiency to participate in an interview. Individuals were excluded if they had cognitive impairment that would impact informed consent or participation, or if they were acutely unwell at the time of the clinic visit.

### Sampling

Where there were multiple willing participants, purposive sampling was applied using predefined criteria established prior to recruitment, including age, gender, AF characteristics (newly diagnosed vs established), and anticoagulation status, as seen in Table 1. This approach ensured a diverse sample of patients attending the AF clinic during the data collection period and helped minimise selection bias. The target sample size was 15 participants, consistent with expectations for achieving data saturation in single-stakeholder qualitative studies [20].

**Table 1:** Patient demographics

	n = 15
*Gender*	
Male	7
Female	8
*Age*	
65–69	2
70–79	8
80–89	3
90+	2
*Duration of AF diagnosis*	
< 1 year	4
1–5 years	9
> 5 years	2
*AF clinic attendance*	
New patient	5
Return patient	10
*Anticoagulant pharmacotherapy*	
Direct Oral Anticoagulant (DOAC)	15
*Rate-control medication use*	
Beta blocker	13

### Recruitment

All patients attending the clinic received a Participant Information Leaflet (PIL) and consent form by post in advance of their scheduled appointment. As part of the clinic’s routine pre-assessment phone call, the nurse confirmed that patients had reviewed the study materials and enquired about their interest in participating. On the day of the clinic visit, healthcare professionals involved in routine care (authors EM and KB) acted as gatekeepers and confirmed suitability and interest in participation. Written informed consent was obtained prior to participation in the interview. New patients to the clinic were interviewed only after AF diagnosis was confirmed, and all participants had seen the clinic pharmacist on the day of interview.

### Data collection

In-depth, face to face semi-structured interviews were conducted in March–May 2025 by the researcher (MD). Interviews took place in a private room within the hospital and lasted approximately 15–30 min. Due to the variable and unpredictable nature of clinic scheduling, interviews were conducted opportunistically on the day of attendance rather than being pre-scheduled. Participants were interviewed one-to-one, and all were offered the opportunity to ask questions before and after the interview. There was no relationship established between the researcher (MD) and participants prior to study commencement or recruitment. A semi-structured topic guide, informed by existing literature, was trialled with two patients in TUH (see Supplementary Material 1). Following the pilot, minor revisions were made to improve clarity and to rephrase questions into a more open-ended format, facilitating richer and more patient-led responses. Data from the pilot interviews were not included in the final analysis. All interviews were audio-recorded, encrypted, transcribed verbatim by a General Data Protection Regulation (GDPR)-compliant transcription service, and anonymised prior to analysis. The transcripts were checked for accuracy by the researcher (MD).Transcripts were not returned to participants for comment or correction due to time constraints and the decision to minimise participant burden, particularly given the older age profile of the study population. In addition, anonymised patient demographic data were collected, including gender, age, duration of AF diagnosis, AF-related medications, and clinical characteristics relevant to their outpatient visit. Collection of these data was included in the informed consent process. Demographic information was obtained directly from participants during the interview, and where details were unclear or unknown, this was supplemented using patient medical records.

### Data analysis

Interviews were transcribed verbatim, anonymised, and analysed using Braun and Clarke’s six-step thematic analysis framework; (1) familiarisation with the data, (2) generating initial codes, (3) searching for themes, (4) reviewing themes, (5) defining and naming themes, and (6) producing the final report [21]. An inductive approach was adopted, allowing themes to develop from the data without being shaped by pre-existing theories or frameworks. To enhance rigour, the primary researcher (MD) manually coded all transcripts, 6 interviews being independently reviewed by two supervisors (CMcA and AS) to ensure consistency and support thematic saturation. As the primary researcher is a clinical pharmacist, coding and theme development were discussed iteratively with supervisors to minimise potential bias and reduce the influence of a single researcher’s interpretation. Themes were developed iteratively and refined through regular consultation with the academic supervisor.

### Ethics approval

Ethical approval was granted by the Tallaght University Hospital/St James’s Hospital Joint Research Ethics Committee (Submission Number 4056: Date Approved: 12/2/25).

## Results

Fifteen interviews were conducted with adults aged ≥ 65 years attending the TUH AF clinic as seen in Table 1. Data sufficiency was reached without new codes emerging after 12 interviews; subsequent interviews added depth but did not generate additional codes. Table 2 presents the identified five overarching themes and associated subthemes.

**Table 2:** Themes and subthemes identified from participant interviews

Theme	Subthemes
1. Understanding of AF	Clarity of information received from health care professionals (HCPs)
	Impact on daily life
	Understanding of stroke risk associated with AF
2. Knowledge and Experience with Pharmacotherapy	Recognition of bleeding risk
	Experiences of medication side effects
	Perceived treatment burden or lifestyle adjustments
	Knowledge of prescribed AF medicines
3. Satisfaction with Care	General satisfaction with AF care
	Perceptions of GP role
	Shared decision-making
4. Role of the Pharmacist in AF Care	Value of AF clinic pharmacist interactions
	Perceptions of community pharmacy role
	Difficulty distinguishing hospital pharmacists from other HCPs
5. Views on Pharmacist Prescribing	Pharmacist specialist knowledge of medicine
	Confidence in pharmacists’ training and education
	Doctor-only prescribing preferred due to established relationships

### Theme 1: Understanding of AF

Overall, patients’ knowledge of AF appeared to be shaped largely by the explanations given by healthcare professionals, though the depth of understanding varied widely. While participants consistently framed AF as a heart-related problem, their insights into rhythm disturbance and awareness of stroke risk were less common.

All participants recognised AF as a heart condition, though the depth of understanding varied widely. Many described it simply as “the heart not working properly” or “an irregular heartbeat,” reflecting a basic awareness of rhythm disturbance.

One participant explained, *“All I know it’s the heart doesn’t stay in a regular rhythm… it tries to make sure your heart doesn’t go bananas*” [P2] Another noted, *“Basically your heart has gone out of sync, the rhythm is not correct. It’s beating too fast”* [P5].

While some could articulate the electrical nature of AF, describing it as *“an electrical current to the heart… it goes a bit out of kilter”* [P8], others admitted they had *“absolutely nothing”* [P13] explained at diagnosis and felt unclear about what AF meant.

Most instead framed the seriousness of AF around how it made them feel day-to-day, such as palpitations, tiredness, or breathlessness, with one saying, *“If you are walking anywhere now, I find I get short of breath… I can’t just keep walking”* [P7].

Few participants connected AF with stroke risk. Only two explicitly described anticoagulation as stroke prevention: *“That particular one was recommended because… it gives you a better chance of not having a stroke”* [P10].

### Theme 2: Knowledge and experience with pharmacotherapy

Overall, participants showed strong awareness of anticoagulant therapy and its risks, with positive attitudes towards adherence. However, their knowledge of rate-control medicines was inconsistent, and very few demonstrated understanding of the specific role of beta blockers in AF management.

Participants consistently demonstrated awareness of the bleeding risk associated with anticoagulant therapy. Many could recall being advised to take care with cuts and bruises, and this message appeared to be well retained.

One participant reflected, *“They did say to watch that if you have any bumps or scratches… they told me that”* [P4].

Another described, *“I know it’s a blood thinner… if I cut myself, it would bleed a bit more”* [P10].

Despite this awareness, few participants had experienced side effects or significant bleeding episodes. Those who did described only minor issues such as nosebleeds or bruising, which were easily self-managed at home. As one participant explained, *“Just the odd nosebleed if I blow too hard, but nothing to worry about”* [P2]*. *No one reported discontinuing or switching treatment because of side effects, and all expressed satisfaction with remaining on anticoagulation, *“I haven’t really had a problem with it”* [P1].

Importantly, participants emphasised acceptance of anticoagulant therapy, viewing it as essential to their AF management. No one expressed disappointment about needing this medication, nor did they describe it as a burden or something that altered their lifestyle. As one participant noted, *“It doesn’t bother me, I just take it every day… I’m happy to do it”* [P11].

While knowledge of anticoagulants was strong, participants were less confident about other AF medicines. All recognised they were taking an anticoagulant for AF, but only around half of those prescribed a beta blocker could identify they were taking two medicines for the condition. Understanding of the beta blocker’s function was limited, with several unsure why it had been prescribed. One explained, *“I know the blood thinner, but I’m not too sure why I’m still on the Bisoprolol”* [P9]. Another explained, *“I just take it because the doctor said… I wouldn’t know what it does”* [P12].

### Theme 3: Satisfaction with care

Overall, participants expressed high levels of satisfaction with the care they had received for AF. Most described a high standard of care received in the AF clinic, and no participants suggested changes or improvements were needed. Only one person noted frustration with the time it had taken to access specialist care, explaining, *“It took nearly two years before I had the ablation done… complete madness”* [P13]. Aside from this exception, participants’ accounts reflected confidence in and gratitude for the care provided.

When discussing their broader care, many participants referred to the role of their general practitioner (GP). The majority described positive experiences, often highlighting trust and a longstanding relationship: *“My GP is great, I’ve been with him years”*[P2]. However, some also reported challenges in accessing timely appointments: *“They’re very good, but sometimes it’s hard to get in when you need to”* [P15].

A strong desire for shared-decision making was not apparent across the interviews, with many participants adopting a more passive stance. Several described being content to follow the advice of their healthcare team, with one saying, *“I just do what they tell me, they know best”* [P5]. Nonetheless, some participants indicated they valued being involved, or felt included in past treatment decisions: *“They explained it all to me and I agreed with the plan”* [P10]. One participant raised concerns about how older people’s views are treated in healthcare more generally, remarking, *“The problem with older people is they’re not listened to”* [P10].

Participants’ accounts suggest a strong sense of satisfaction with AF care, trust in their GPs and clinic team, and limited desire for changes to current services. While SDM was not universally prioritised, some patients clearly valued opportunities to contribute to their treatment decisions.

### Theme 4: Role of the pharmacist in AF care

Despite direct interaction, participants often did not recognise that they had spoken with the pharmacist in the AF clinic. The pharmacist was frequently confused with other members of the clinical team, such as nurses or doctors. As one participant admitted, *“I didn’t know what a pharmacist was until then”* [P7]. Another reflected, *“I thought she was one of the doctors”* [P12].

However, once it was clarified who the pharmacist was, all participants spoke positively about their experiences. Many valued the time taken to listen, explain medicines, and provide reassurance. Several recalled specific interventions, including being advised on the correct timing of their direct oral anticoagulant (DOAC), receiving an alert card, and being reminded to inform other healthcare professionals about their anticoagulant use. One participant described, *“She told me to take it twelve hours apart, and I’ve done that ever since”* [P4]. Another said, “*They gave me a card to carry… I thought that was a good idea”* [P14].

Although not the focus of these interviews, participants frequently mentioned their community pharmacists, and these reflections were consistently positive. Community pharmacists were seen as approachable and accessible, with one participant commenting, “They’re always very good in the chemist, if I ask, they’ll explain” [P15]. The prominence of community pharmacy in primary care, and its familiar role, meant that when “pharmacist” was mentioned, participants tended to think first of their community pharmacist. By contrast, the role of hospital-based clinical pharmacists was less clearly defined and less readily recognised. While many had encountered a hospital pharmacist in clinic and during inpatient stays, the community pharmacist role was easier to identify, underscoring the relative invisibility of clinical pharmacists in outpatient settings.

Many participants did not initially recognise that they had spoken with the pharmacist in the AF clinic, often confusing them with doctors or nurses. Once identified, pharmacists were described as approachable and informative, providing practical medicines advice and reassurance. Community pharmacists were more readily recognised and frequently mentioned as accessible and helpful, while the hospital pharmacist’s role was less recognisable to patients.

### Theme 5: Views on pharmacist prescribing

Participants expressed mixed but generally cautious views on the idea of pharmacists taking on prescribing responsibilities in AF care. Many highlighted pharmacists’ specialist knowledge of medicines as a reason they could be trusted to prescribe. One participant remarked, *“It’s what they’ve studied, they know the ins and outs of the tablets”*. [P10] Others described pharmacists as highly knowledgeable and detail-focused, suggesting that this expertise could support safe prescribing decisions, *“You’d imagine they’d be more inclined to know more about the tablets than the doctor would*” [P11] and “*You’re the people with the knowledge of it you know”* [P1].

Some participants emphasised the need for reassurance that pharmacists had the appropriate training to take on prescribing roles. While they acknowledged pharmacists’ medication knowledge, they wanted confirmation that this was supported by formal education and treatment planning. As one participant reflected, *“If they’ve been trained properly, then yes, I would be happy with them prescribing”* [P5].

Despite recognising pharmacists’ skills, strong trust in doctors was evident, and several participants were hesitant to shift prescribing responsibilities away from physicians. For some, doctors remained the only professionals they were comfortable with in relation to prescribing authority. One participant explained, *“I’d go with the doctor, you know… they’re the ones who should make that call” [P11]*. Another added, *“Pharmacists know a lot, but at the end of the day, I’d rather the doctor decide”* [P15].

Overall, while participants respected pharmacists’ expertise in medicines and many were open to the idea of pharmacist prescribing if appropriate training was assured, deep-rooted trust in doctors continued to shape their views.

## Discussion

This qualitative study explored patients’ views on AF management and the pharmacist’s role within a multidisciplinary AF clinic in an Irish hospital. Fifteen older adults were interviewed about their understanding of AF, medicines, and care experiences. Participants showed good awareness of anticoagulant therapy and bleeding risk but limited understanding of stroke prevention and rate-control medicines. Satisfaction with care was high, though most took a passive role in decisions. Clinical pharmacists were viewed positively once recognised, yet their role was often unclear. Views on pharmacist prescribing were mixed; with some being supportive, while others retained greater trust in doctors.

This study shows a key gap in atrial fibrillation care: while most patients know AF is a heart problem and reliably take anticoagulants, many still do not understand their stroke risk, overall treatment goals, or the role of other medicines [22–24]. Our findings mirror the EHRA survey, which reported persistent gaps in patients’ understanding of their diagnosis and medicines, especially difficulty identifying which drugs are for AF and why they are needed [7, 25]. In our cohort, awareness of bleeding risk from anticoagulation was strong and retained, but knowledge of rate-control therapy (e.g. beta-blockers) was inconsistent. This pattern likely reflects where educational emphasis currently falls in the clinic: anticoagulation is rightly treated as high-risk and receives focused counselling, with the pharmacist providing written information on anticoagulants and alert cards specific to individual DOACs, while other aspects of AF management may receive less attention within time-limited encounters [26, 27].

The implications are important. Stroke literacy requires strengthening. Although most participants were content with their care and medication plans, few spontaneously linked AF to stroke risk or described warning symptoms. Given that stroke treatment is time-critical within the first few hours of symptom onset, a lack of risk awareness and symptom recognition may delay presentation and reduce eligibility for reperfusion therapy [28]. Enhancing education on stroke risk, FAST symptoms (facial drooping, arm weakness, speech disturbance, and the need for urgent action), and what to do if symptoms occur should be a core, repeated element of AF care, alongside anticoagulant counselling [12, 29].

Although SDM is recognised as best practice in AF care, the results from this study show it remains poorly embedded in routine care [4, 5]. Participants were generally very satisfied with their AF care but still tended to adopt a passive stance, trusting clinicians to decide despite evidence that SDM improves adherence and satisfaction [4, 30]. For older adults managing multimorbidity and polypharmacy, SDM can be made practical through structured prompts and teach-back, concise decision aids focused on stroke prevention versus bleeding trade-offs, and personalised risk framing (e.g. absolute stroke risk reduction with anticoagulation) [6, 31–33]. Embedding these tools into clinic consultations would align with 2024 ESC guidance on integrated, patient-centred care and could help translate high-level recommendations into routine practice [34, 35].

Pharmacist visibility and role clarity need attention. Many patients equated “pharmacist” with their community chemist, while the hospital-based pharmacist was less visible and often mistaken for a doctor or nurse. Once informed they had met the pharmacist, participants expressed high satisfaction with the care received. This lack of recognisability is not unique to our site and has also been reported in hospital outpatient clinics in Spain, where patients were satisfied once they understood the pharmacist’s role but initially unaware of their involvement [36]. Simple operational fixes (“meet your AF team” handout, and consistent documentation of pharmacist input in clinic letters) could improve recognition and, by extension, patient trust and recall of advice [36]. Clear role recognition allows patients to know whom to approach with medication questions or side effects and reduces confusion about medication related follow-up.

The introduction of an ASP in cardiology could broaden this remit from “high-risk medicine counselling” to “whole-condition medicines optimisation.” ASPs, by design, work at an expert level across complex pharmacotherapy [17]. Based on models implemented in other countries such as Canada, an AF clinic could incorporate pharmacist-led systematic education, polypharmacy review, proactive dose checks, deprescribing, and structured SDM [37–44]. Creating an ASP-led cohort clinic e.g. for patients with multimorbidity, polypharmacy and recent medication changes would target those most likely to benefit while preserving medical consultant and ANP capacity for procedural and diagnostic complexity [45, 46]. Although TUH does not currently have a cardiology ASP, similar posts exist elsewhere in Ireland; adopting such a role locally would be consistent with national direction and international advanced practice frameworks [46, 47]. These roles are typically supported by structured postgraduate education (e.g. MSc-level clinical pharmacy training), ongoing continuous professional development (CPD), and competency frameworks aligned with national and international advanced practice standards [48].

Participants’ views on the potential of pharmacist prescribing were cautiously supportive, anchored in recognition of pharmacists’ specialist medicines knowledge but still having a high level of trust in doctors [5, 49–51]. This is unsurprising in an older cohort and in an Irish context where pharmacist prescribing is not yet routine. Nonetheless, this activity is expanding globally, with countries such as the United Kingdom, United States, Canada, Australia, Poland, Switzerland, and Denmark now permitting independent pharmacist prescribing [47]. This was most commonly in community or primary care or within collaborative care models [13, 52]. In the Irish context, these findings are particularly relevant because pharmacist prescribing is now beginning to expand in community practice through the Common Conditions Service, while broader implementation across the health service remains in development. Our findings therefore suggest that patient trust, education, and understanding of the pharmacist’s role will be central to the acceptability of any future expansion of pharmacist prescribing into specialist outpatient settings such as AF clinics. Overall, while participants in this study were open to pharmacist prescribing if appropriate training and oversight were assured, deep-rooted trust in doctors continued to shape their views. Clear communication about the pharmacist’s prescribing role will be essential to maintain patient confidence and trust [12, 13, 53, 54].

By leveraging pharmacists’ unique whole-regimen expertise and formalising that capability through an ASP model, TUH can move from excellent anticoagulant counselling to consistently excellent AF care literacy, without losing sight of safety. In doing so, the service would better align with contemporary guidelines, close the understanding–adherence loop, and potentially improve time-critical stroke outcomes for patients living with AF. These findings have important implications for policy development in Ireland, where the expansion of pharmacist prescribing is currently being considered. Integrating pharmacists into established care pathways, such as AF clinics, may also support gradual acceptance by reinforcing their role within a familiar, collaborative model of care which aligns to policy such as that in the UK [55].

### Strengths and weaknesses

This study provides valuable insight into the experiences of patients living with AF, for whom anticoagulation and medicines management are central to care. Semi-structured interviews enabled participants to express their views freely, generating rich qualitative data that captured both understanding and attitudes toward pharmacists and SDM. Braun and Clarke’s thematic analysis offered a systematic and transparent approach, enhancing rigour and credibility. Importantly, the study explored a relatively under-researched area in Ireland: patients’ perspectives on the pharmacist’s role in AF management.

However, the study’s scope was limited to a single hospital-based AF clinic, which may restrict transferability to other settings. All participants were aged ≥ 65 years, so perspectives of younger patients were not captured. Recall bias may have influenced accounts of earlier interactions, and participation within the clinic setting could have reduced willingness to share criticism. The researcher’s dual role as a clinical pharmacist may also have introduced unintentional bias.

## Future research

Future research could build on these findings by exploring the perspectives of other key stakeholders in AF care. Interviews with medical consultants, non-consultant hospital doctors (NCHDs), and advanced nurse prescribers working in AF clinics would provide valuable insight into how the pharmacist’s role and potential for prescribing are perceived within the wider multidisciplinary team. Understanding these views would help identify opportunities and barriers to expanding pharmacist-led services.

## Conclusion

This study highlights strong engagement with anticoagulant therapy among older adults with atrial fibrillation but persistent gaps in understanding of stroke risk and rate-control treatment. Satisfaction with care was high, yet most participants adopted a passive role in decisions, indicating that SDM remains under-realised. Hospital pharmacists were valued once recognised, but their role was often unclear, mirroring findings internationally. Improving pharmacist visibility and embedding structured education on stroke prevention could enhance patient understanding and confidence.

An ASP model embedded within the clinic could potentially lead medicines optimisation and pharmacist prescribing. This would streamline care, enhance prescribing safety, and ensure consistent, expert input for complex AF patients.

## Supplementary Information

Below is the link to the electronic supplementary material.

Supplementary file1 (DOCX 18 KB)

## Data Availability

The datasets generated and analysed during the current study are not publicly available due to the qualitative nature of the data and the potential risk of participant identification, particularly given the small sample size and single-site setting.
