# Supplementary material for: Patient perspectives on atrial fibrillation management and pharmacist roles in outpatient clinics: a qualitative study
Source: Int J Clin Pharm. 2026 Apr 29;48(5):1840–9. doi: 10.1007/s11096-026-02155-7 (PMC13569569; doi:10.1007/s11096-026-02155-7)
Supplement: Supplementary file 1 [file 11096_2026_2155_MOESM1_ESM.docx]

**Appendix 1:**

| **COREQ 32-Item Checklist – Reporting of Study Methods and Findings** | | |
| --- | --- | --- |
| **Domain / Item** | **Guide Question** | **Reported in this study** |
| **Domain 1: Research team and reflexivity** |  |  |
| 1. Interviewer/facilitator | Which author conducted the interviews? | MD (primary researcher, MSc student) |
| 2. Credentials | What were the researcher’s credentials? | MPharm, MPSI (hospital pharmacist, MSc candidate) |
| 3. Occupation | Occupation at time of study | Hospital pharmacist, MSc Hospital Pharmacy student |
| 4. Gender | Male/female | Female |
| 5. Experience and training | Relevant training | Trained in qualitative methods as part of MSc; supervised by academic & hospital supervisors |
| 6. Relationship established | Was a relationship established prior to study? | No relationship established before recruitment |
| 7. Participant knowledge of interviewer | What did participants know? | Knew MD was a pharmacist researcher; informed of study aims via PIL |
| 8. Interviewer characteristics | Bias/assumptions/interests | Pharmacist interest in AF management and pharmacist role development |
| **Domain 2: Study design** |  |  |
| 9. Methodological orientation | Underpinning theory | Thematic analysis (Braun & Clarke, 2006), inductive approach |
| 10. Sampling | How selected? | Purposive sampling |
| 11. Method of approach | How approached? | Postal invitation + nurse pre-assessment phone call + in-clinic confirmation |
| 12. Sample size | Number of participants | 15 participants (target achieved) |
| 13. Non-participation | Refusals/drop-outs | Not systematically recorded; only those consenting participated |
| 14. Setting of data collection | Where? | Private room in Tallaght University Hospital AF clinic |
| 15. Presence of non-participants | Anyone else present? | No |
| 16. Description of sample | Key characteristics | Adults ≥65 with AF; demographic and clinical details collected |
| 17. Interview guide | Guide provided/piloted? | Semi-structured topic guide developed from literature; piloted with two patients |
| 18. Repeat interviews | Any repeat? | No |
| 19. Audio/visual recording | Recorded? | Yes, audio-recorded and transcribed verbatim |
| 20. Field notes | Made? | No formal field notes |
| 21. Duration | Interview length | 15–30 minutes |
| 22. Data saturation | Discussed? | Yes, aimed for saturation; 15 participants considered sufficient |
| 23. Transcripts returned | To participants? | No, due to minimising burden |
| **Domain 3: Analysis and findings** |  |  |
| 24. Number of data coders | How many? | One (MD), with subset reviewed by supervisors (CMcA, AS) |
| 25. Description of coding tree | Provided? | Not a full coding tree, but themes described |
| 26. Derivation of themes | Predefined or emergent? | Emergent (inductive) |
| 27. Software | Used? | No. Manual coding reported |
| 28. Participant checking | Feedback on findings? | No |
| 29. Quotations presented | Illustrative quotes? | Yes, anonymised with participant ID |
| 30. Data and findings consistent | Consistency? | Yes, findings supported by data |
| 31. Clarity of major themes | Major themes clearly presented? | Yes |
| 32. Clarity of minor themes | Minor/dissenting views reported? | Yes, diverse cases acknowledged |

**Appendix 2**

**Topic Guide:**

The interview will focus on the following key areas:

1. **Patient's Understanding of Atrial Fibrillation and Its Management**
   - Explore their knowledge of AF and its impact on their health.
   - Understand their experience with managing the condition, especially regarding anticoagulation therapy.
2. **Pharmacist Involvement in Their Care**
   - Assess how patients perceive the pharmacist's role in their AF management.
   - Explore specific pharmacist-led interventions (education, medication management) they may have experienced.
3. **Shared Decision-Making**
   - Evaluate how involved patients feel in the decision-making process, particularly regarding their treatment options.
4. **Satisfaction and Outcomes**
   - Gather insights on patient satisfaction with care and how pharmacist interventions may have affected outcomes such as adherence or understanding of treatment.
5. **Barriers to Effective Management**
   - Identify any challenges or barriers patients face in their AF management and where pharmacist support might be lacking or could be improved.
   - Pharmacist prescribing – future of the role.

**Interview Questions – Patient Perspectives on AF Care**

**Question 1**
*When were you first diagnosed with atrial fibrillation, and how was it identified?*
**Prompt:** Who informed you of the diagnosis? Was the condition explained to you at that time?

**Question 2**
*In your own words, what is your understanding of atrial fibrillation?*
**Prompt:** How do you think AF affects your health?

**Question 3**
*How many medicines are you taking for atrial fibrillation, and what do you understand about what each one does?*
**Prompt:** What do you think the blood thinner is for? What about the rate-control medication?

**Question 4**
*Can you tell me about your experience of taking your AF medicines, including any advice you were given about safety or side effects?*
**Prompt:** Have you ever received information about bleeding risk or what to do if side effects occur?

**Question 5**
*Do you feel you were given enough information and support when you were first diagnosed or when your medicines were started?*
**Prompt:** Who provided that information (GP, nurse, pharmacist, hospital team)? Would you have liked more or in a different format?

**Question 6**
*Have you met a pharmacist as part of your AF care in this clinic or elsewhere? How did you find that interaction?*
**Prompt:** Were you aware that a pharmacist was part of the AF clinic team before today?

**Question 7**
*Is there anything about how the clinic is run, or how information is given, that helps or makes it harder to manage your AF or medicines?*
**Prompt:** If you could change one thing about how information or support is provided, what would it be?

**Question 8**
*How do you feel about your role in decisions about your AF treatment? Do you prefer to make decisions together or trust your healthcare team to decide?*
**Prompt:** Do you feel involved, or do you prefer to be guided by the professionals?

**Question 9**
*In Ireland, pharmacists may soon be allowed to prescribe in clinics like this. How would you feel about a pharmacist prescribing your AF medicines?*
**Prompt:** What would help you feel confident in that, or what concerns might you have?

**Question 10 (Closing)**
*Is there anything else you would like to add about your experience of AF care, medicines, or your interaction with the clinic team?*
**(Thank participant and end recording.)**
